# Chloroform Anesthesia and Narcosis as a Remedy in Cerebro-Spinal Meningitis

**Published:** 1888-12

**Authors:** C. H. Harris

**Affiliations:** Cedartown, Ga.


### CHLOROFORM ANESTHESIA AND NARCOSIS AS A REMEDY IN CEREBRO-SPINAL MENINGITIS.

BY C. H. HARRIS, M. D., CEDARTOWN, GA.

The duties of a doctor are four-fold. His first are to his patients, next to humanity, next to his profession and the last to himself. When, in the pursuit of his profession, he thinks he has observed a new fact of interest, he should hasten to give to the profession and humanity the benefit of his observations though he may be the sufferer. It is with this motive that, I endeavor to arrest your attention, while in doing so I am compelled to make a record of a mistake. After all, our blunders are our best teachers. While they grind deep the wrinkles of experience, they make indelible impressions on our memory. They are common to all—more especially to the physician whose arduous calling and varied studies open pitfalls that are unknown in other pursuits.

The lesson which I have drawn from a recent mistake is the possibility, or I should say, strong probability that we have in chloroform a valuable remedy in cerebro-spinal meningitis.

For several years the physicians of this section have encountered but very few cases of this dreadful malady. Indeed, so free have we been from it that in approaching the bedside the subject rarely entered our minds. Last month I encountered a case, which I now report, as it will serve to illustrate the value of chloroform in its treatment, though the case terminated in death.

Willie V., aged seven years, a boy of extraordinary sprightliness of mind and remarkable precocity, was taken quite ill on the 11th ult. with something like a light convulsion, a very severe headache and a strange affection of the eyes. They were drawn downward to the extent of hiding both corners behind the lower lids. There was slight stiffness of the dorsal muscles. No fever, as tested by the thermometer under his tongue and arm. Respiration and pulse normal. Mind perfectly clear. In fact, none other but the nervous symptoms. In a few minutes, however, he had a "spell," which was so queer that I was led to believe that his sexual organs were involved in the trouble. Suspecting masturbation as a factor, in the height of the paroxysm I suddenly threw off the cover and revealed an array of symptoms that I have never seen before and which led me to believe I had discovered the key to his case. There was his penis of a size proportionate to one twice his senior in a state of intense erection and his frame trembling as if thrilled by an orgasm. It terminated in a long and sharply phymosed prepuce which stretched tightly over the glans. There was also a luxurious growth of hair an inch long and remaining sexual apparatus giving evidence of remarkable precocity. Boy only 7 years old with the sexual organs of a lad of 16 years—no fever, pulse good, mind clear and giving intelligent and prompt answers to all questions. Perhaps such experts as Dr J. Lewis Smith or Dr. Wilson might have seen enough in this case to pronounce it meningitis from the start. I am quite sure, however, that Dr. Sayre or Dr. Keys would have regarded the profound nervous disturbances as the result of congenital phymosis and adhesions and depraved sexual habits, and that circumcision would cure him. This was the view I took of the case, and it is proper to add that I was sustained in it by two educated physicians of established reputation and experience. The family were told our opinion; the boy, who was perfectly rational, gave his consent to the operation. We lost no time in chloroforming and circumcising him. As soon as he came under the chloroform, which was pushed to complete anesthesia, every symptom disappeared as if by magic, and we had no fears or anxiety about our patient for five days. The wound was almost healed and the boy, as we thought, in a fair way for recovery. Imagine my consternation and surprise when, on the evening of the fifth day, I was hurriedly called to him and immediately recognized the real nature of the case. Wild delirium, flaming eyes more tensely drawn than ever, extreme opisthotonus, head and heels nearly touching, labored breathing, elbows drawn back, hands clenched, high and varying temperature, rapid and irregular pulse—in a word, all the array of nervous symptoms in full force and stamping it a typical case of meningitis. For five days the disease was in mask; now it was uncovered and the truth revealed. The delirium was supplanted by coma, which rapidly deepened, and in two days ended in death. In this short time destructive inflammation of one eye took place, resulting in perforation of the cornea and shrinking of the ball.

The point in this case I ask you to note is the remarkable change wrought by the chloroform. It was a case of meningitis from the beginning. *The chloroform undoubtedly arrested its progress* for nearly five days. Had I diagnosed correctly at first and repeated the chloroform *inhalations* daily to the extent of producing several hours of sleep each day, I have a deep conviction the result would have been different.

In the last thirty years, in military and civil practice and among the convicts I have had several emergency cases attended with opisthotonus and have invariably used chloroform. In none were the results bad. They gave me no concern and I thought nothing about them then. But since treating this case it occurs to me I might have aborted some cases of meningitis. Is there any form of headache that chloroform will not relieve—any kind of convulsion it will not mitigate? In meningitis are the membranes hyperæmic? What better remedy than chloroform? Ergot is as nothing compared with its depleting effects on the brain. Indeed, it would seem theoretically, as happened in the case I report, that chloroform would fulfill every indication of treatment. True, there is danger in the remedy, but it pales in view of the hundred-fold greater danger of the disease. So often during the war did I observe its salutary effects in traumatic fever that I have been eager to try it in idiopathic fevers. Supplemented with an opiate, chloroform is the remedy for a chill, be the cause what it may.

The treatment of meningitis, with due deference to our teachers, amounts practically to nothing. Powerful remedies have been used with little if any improvement upon the past. It stands thus: if the epidemic is mild, some cases get well under varied and opposite treatment. If the epidemic is malignant, all die exc[illegible]t a few who are seriously damaged for life. Is there not r[illegible] for a change? Does not the clinical history of the case I have just reported admit one ray of light into the dark abyss, w[illegible] [illegible]oaning depths have never yet felt the touch of science?

